# Supplementary material for: Causal Relationship between Aging and Anorexia Nervosa: A White-Matter-Microstructure-Mediated Mendelian Randomization Analysis
Source: Biomedicines. 2024 Aug 16;12(8):1874. doi: 10.3390/biomedicines12081874 (PMC11351342; doi:10.3390/biomedicines12081874)
Supplement: Supplementary file 1 [file biomedicines-12-01874-s001.zip › Supplementary Figure/Figure S8/Leaveoneout plot of MR analysis for TL to ubm-b-2021.pdf]

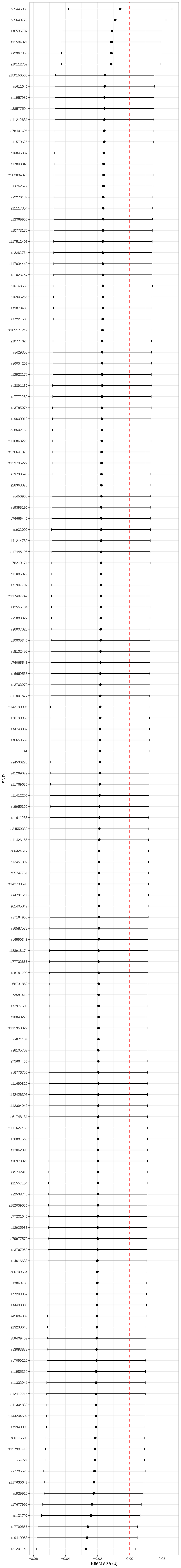

rs35446936
rs35640778
rs6536702
rs11584821
rs2967355
rs10112752
rs150150565
rs611646
rs1957937
rs28577594
rs11212631
rs78491606
rs11579626
rs10845387
rs17803849
rs202034370
rs762679
rs2276182
rs11117354
rs12369950
rs10773176
rs117512405
rs2282764
rs117034449
rs1023767
rs10768683
rs10905255
rs9878436
rs7221585
rs185174247
rs10774624
rs429358
rs6054257
rs12932179
rs3891167
rs7772289
rs3785074
rs9600019
rs28502153
rs116863223
rs376641875
rs139795227
rs73730598
rs28363070
rs450962
rs9398196
rs76666449
rs932002
rs141214782
rs17445108
rs76219171
rs11085072
rs1907702
rs117407747
rs2555104
rs1003322
rs6007020
rs10805346
rs8102497
rs76065543
rs6669563
rs2763979
rs11991877
rs143190905
rs6790988
rs4743037
rs6659669
All
rs4530278
rs41269079
rs11769630
rs11412296
rs9955360
rs1611236
rs34550383
rs11426156
rs80324517
rs12451892
rs55747751
rs142730696
rs4731541
rs61405042
rs7164950
rs6587577
rs6590343
rs188918174
rs77732866
rs6751209
rs66731853
rs73581419
rs2977608
rs10840270
rs111950327
rs871134
rs8105767
rs75664430
rs6776756
rs11699829
rs142426306
rs112394943
rs61748181
rs111527438
rs6881568
rs13062095
rs16978028
rs5742915
rs11557154
rs2538745
rs182059586
rs77231040
rs12925933
rs79977579
rs3767952
rs4616688
rs56799554
rs869785
rs7209057
rs4498805
rs45604339
rs13230646
rs59409453
rs3093888
rs7099229
rs1985369
rs1332941
rs12412214
rs41304832
rs144204502
rs9940099
rs80116508
rs137901416
rs4724
rs7705526
rs117630647
rs939916
rs17677991
rs131797
rs7790856
rs9419958
rs1291143
SNP
−0.06
−0.04
−0.02
0.00
0.02
Effect size (b)
